# Controllable beam reshaping by mixing square-shaped and hexagonal optical vortex lattices

**DOI:** 10.1038/s41598-019-38608-5

**Published:** 2019-02-14

**Authors:** L. Stoyanov, G. Maleshkov, M. Zhekova, I. Stefanov, G. G. Paulus, A. Dreischuh

**Affiliations:** 1 Department of Quantum Electronics, Faculty of Physics, Sofia University, 5, J. Bourchier Blvd., Sofia, 1164 Bulgaria; 2 Institute of Optics and Quantum Electronics, Friedrich Schiller University, Max-Wien-Platz 1, D-07743 Jena, Germany; 3 Helmholtz Institute Jena, Helmholtzweg 4, D-07743 Jena, Germany

## Abstract

In the present work we show experimentally and by numerical calculations a substantial far-field beam reshaping by mixing square-shaped and hexagonal optical vortex (OV) lattices composed of vortices with alternatively changing topological charges. We show that the *small-scale structure* of the observed pattern results from the OV lattice with the *larger* array node spacing, whereas the *large-scale structure* stems from the OV lattice with the *smaller* array node spacing. In addition, we demonstrate that it is possible to host an OV, a one-dimensional, or a quasi-two-dimensional singular beam in each of the bright beams of the generated focal patterns. The detailed experimental data at different square-to-hexagonal vortex array node spacings shows that this quantity could be used as a control parameter for generating the desired focused structure. The experimental data are in excellent agreement with the numerical simulations.

## Introduction

Due to the spiral phase profiles of their wavefronts, optical vortices (OVs) are the only known truly two-dimensional (2-D) singular beams^1^. The central singular points of these spiral phase ramps have an undefined phase. Therefore the intensity must decrease to zero at these points, leading to a characteristic ring-shaped beam profile^2–4^. OVs carry photon orbital angular momentum, which can be transferred to matter^5,6^. This angular momentum is proportional to the topological charge (TC) *m* - an integer number with sign, corresponding to the total phase change 2 *πm* over the azimuthal coordinate *φ*. Two singly charged OVs with equal TCs placed on a bright coherent beam experience rotation and mutual repulsion^2,7^. Nesting two OVs of opposite TCs at the same positions result in their translation with respect to the host beam, in their mutual attraction and, eventually, in annihilation. In both self-focusing and self-defocusing third-order nonlinear media, the described interactions remain qualitatively the same during the initial stage of nonlinear evolution^8^. However, the OV dynamics in self-focusing media can be considerably influenced by the reshaping of the neighboring part of the host beam^8^.

By a proper choice of the topological charge of a “control-OV” nested in the ensemble center one can stabilize ensembles of equally-charged OVs against rotation^9^. In^9,10^ this approach is further developed towards large stable regular OV lattices. First experimental results are published in^10^. The stability of square-shaped OV lattices in both self-focusing and self-defocusing nonlinear media is demonstrated in^8^ (Figs. 11 and 12) as well as in linear media^11^. The discrete nondiffracting beams, which are one of the four different families of such beams, are characterized and summarized in^12^ (see also the references therein). Some of the results (e.g. Fig. 2(e,f) in^12^ are closely related to this work. The results in^13^ confirmed that the focal (artificial far-field) patterns of 2-D nonlinear photonic lattices of different symmetries provide the necessary initial conditions for creating optically induced waveguides in photorefractive media. In addition to the intriguing physics involved in OV lattice creation and manipulation, such possible applications (see also^14,15^) as well as the rapidly growing interest in orbital angular momentum multiplexing of information^16^ for data transfer using complex optical fields^17–21^ stimulated the work presented in the following. Last but not least, the trend towards miniaturization resulting in the development of isolated OV emitter based on microring resonator^22^ has recently lead to the demonstration of an on-chip hexagonal OV lattice emitter based on three-plane-wave interference of light coming from parallel waveguides with etched tilt gratings^23^.

The terms lattice constant or lattice node spacing used in this work denote the distance Δ between two neighboring vortices in any vortex lattice or array. The ability to manipulate (add, subtract and, eventually, erase) the TC of an isolated OV or even of a large OV array^24–26^ is an essential part of the physics of the present results. We demonstrate that this is not only valid for a single OV, but also holds for OV lattices (square-shaped and hexagonal) composed of hundreds of OVs on a single background beam. We show that the *small-scale structure* of the observed pattern results from the OV lattice with *larger* array node spacing, whereas the *large-scale structure* comes from the OV lattice with the *smaller* array node spacing. The detailed experimental data at different square-to-hexagonal vortex array node spacings presented in this paper prove that this quantity can be used to control the generation of the desired focused structure. We confirm the ability to host an OV, a 1-D or a quasi-2-D dark beams in each of the bright beams of the far-field pattern. The numerical data are shown to be in excellent agreement with the experimental results.

## Experimental setup and Numerical Simulations

**Figure 1:**
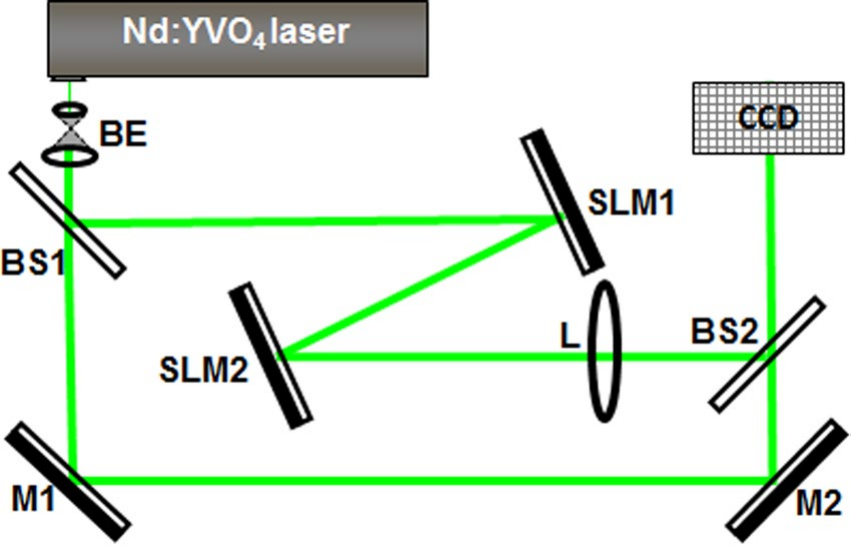
Experimental setup. *Nd*: *YVO*_4_, frequency-doubled continuous-wave Neodymium-doped yttrium orthovanadate laser emitting at a wavelength *λ* = 532 nm; *BE*, beam expander; *M*, flat silver mirrors; *BS*, beam splitters; *SLM*, reflective spatial light modulators (model Pluto, Holoeye Photonics); *L*, focusing lens (*f* = 100 cm); *CCD*, charge-coupled device camera.

The experimental setup is shown in Fig. 1. Pump beam from a continuous-wave frequency-doubled Neodymium-doped yttrium orthovanadate (*Nd*: *YVO*_4_) laser is first expanded by the beam expander *BE* and then illuminates the first reflective spatial light modulator *SLM*1. This SLM modulates the phase of the input Gaussian beam (and, as a consequence, also its amplitude/intensity) and redirects it to a second spatial light modulator *SLM*2 of the same type. The singular beam reflected from *SLM*2 is then focused by a lens *L* (*f* = 100 cm) onto a *CCD* camera chip of 1600 × 1200 pix. (7.2 × 5.4 mm). The *SLM*2-to-lens distance is 95 cm. A reference beam is split off the laser beam before *SLM*1 by a beam splitter (*BS*1). For diagnostic purposes, the object and the reference beams are recombined by a second beam splitter (*BS*2) to interfere at the *CCD* camera chip. Intensity distributions of the resulting optical beams and the respective interference patterns are recorded by the same camera by blocking/unblocking the reference laser beam. *SLM*1 and *SLM*2 are aligned in parallel with a distance of 49 cm. At the SLMs the angle of incidence of the laser beam with respect to the normal incidence is 4°. The efficiency of the single OV lattice generation is 71% ensuring overall efficiency of nearly 45%. The reason to use two SLMs instead of imaging *SLM*1 onto *SLM*2 is to include in the experiment the amplitude modulation resulting from free-space propagation between the modulators and from the second modulator to the lens. According to this choice, in the numerical simulations we considered the evolution of the manipulated light field after each of these elements until the artificial far field is reached. Both the setup and the simulations would simplify considerably when imaging *SLM*1 onto *SLM*2 thereafter Fourier-transforming the plane of *SLM*2. The amplitude and phase modulation could be tailored in first diffraction order of the SLM by applying a suitable weighted blazed grating to the modulator^27^.

Since the propagation of the laser beam in the object arm of the setup is linear (Fig. 1), its evolution was numerically calculated by using the linear paraxial model equation for the slowly-varying optical beam envelope amplitude *E*

1 $$i\partial E/\partial (z/{L}_{Diff})+\mathrm{(1}/\mathrm{2)}{{\rm{\Delta }}}_{T}E=0.$$

Here the transverse part of the Laplace operator is denoted by Δ_*T*_, the diffraction length of an individual OV - by *L*_*Diff*_ = *ka*^2^, and the wave number in air is *k*. In a computational window spanning over 1024 × 1024 points the half width at the 1/*e*^2^ intensity level of the host Gaussian beam was 205 pix. By phase modulation, using *SLM*1, we generated a single OV and recorded its profile right after *SLM*1 and at the position of the second SLM. From the data we concluded that the distance between *SLM*1 and *SLM*2 corresponds to 1.5*L*_*Diff*_ and the distance from *SLM*2 to the lens - to 3.0*L*_*Diff*_. The presence of a lens in the experimental setup was accounted for by the transmission phase function *T*(*x*, *y*) of a thin lens with a focal length *f*

2 $$T(x,y)={\exp }\{\,-\,ik({x}^{2}+{y}^{2})/(2f)\}$$

in the respective plane. The beam evolution was then numerically followed to the focal plane (41.0*L*_*Diff*_ behind the lens).

**Figure 2:**
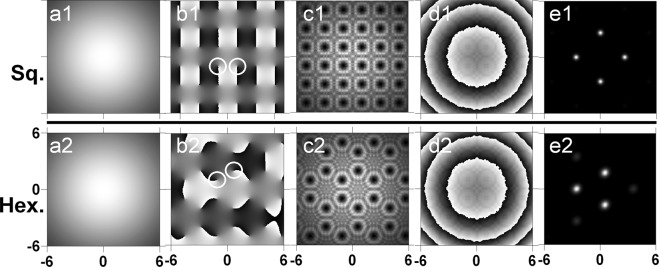
Vortex lattice generation with one SLM. Numerical results for array node spacing 41 pix. Intensity of the background beam (**a1**,**a2**) illuminating *SLM*1, square-shaped (**b1**) and hexagonal (**b2**) phase distributions (with pairs of OVs encircled to underline their opposite TCs) sent to this modulator and respective intensity distributions (**c1**,**c2**) just in front of the focusing lens. The phase used for simulating the action of a focusing lens is shown in frames (**d1**,**d2**). The respective intensity profiles of the sub-beams in the artificial far field for square and hexagonal OV lattice are shown in frames (**e1**,**e2**), respectively. In each frame we show 6% of the total computational area.

In Fig. 2 we show numerical results for the separate creation of a large square-shaped (a1–e1) and a hexagonal lattice (a2–e2) of OVs with alternatively changing TCs. In both cases the array node spacing is 41 pix. In the experiment, separate OV lattice generation means that one of the SLMs is either switched off thus acting as a mirror, or it is programmed with a flat phase distribution. The intensity profile of the background beam illuminating *SLM*1 is shown in frames (a1, a2). The phase distributions sent to *SLM*1 are displayed in the second column, where the phase profile of the square-shaped OV array is shown in panel (b1) and the one of the hexagonal OV array in panel (b2). The pairs of white circles in frames (b1) and (b2) denote two neighboring OVs. Their opposite TCs can be recognized by the fact that the phase of one of the OVs changes from black to white (i.e. from 0 to 2*π*) around the center of the circle in clockwise direction, in the neighboring circle – in counter-clockwise direction. The respective simulated intensity distributions just in front of the focusing lens are presented in frames (c1) and (c2). In both cases, a rotation of the OV lattices is suppressed by the opposite signs of the TCs of neighboring OVs as expected^9,10^. In the plane of the lens the phase profile of the lens (identical frames (d1, d2)) is added, causing the beam to converge into a focus. The resultant intensity profiles of the beams in the artificial far field are shown in the last column of frames in Fig. 2. When a square-shaped OV lattice is encoded on one of the SLMs, the calculated far-field intensity profile (frame (e1)) clearly shows the presence of four peaks situated in the apices of a rhomb. When a hexagonal OV lattice is used on one of the SLMs, the calculated far-field intensity (frame (e2)) is composed by three dominating peaks situated in the apices of an equilateral triangle. This bright peak triangular structure is inscribed in a another less intensive triangular structure of beams. The numerical results obtained for two times more dense lattices (node spacing of 21 pix.) are qualitatively the same. In each frame of Fig. 2 spanning over (−6, 6) arbitrary units 6% of the total computational area is shown.

## Results and Discussion

**Figure 3:**
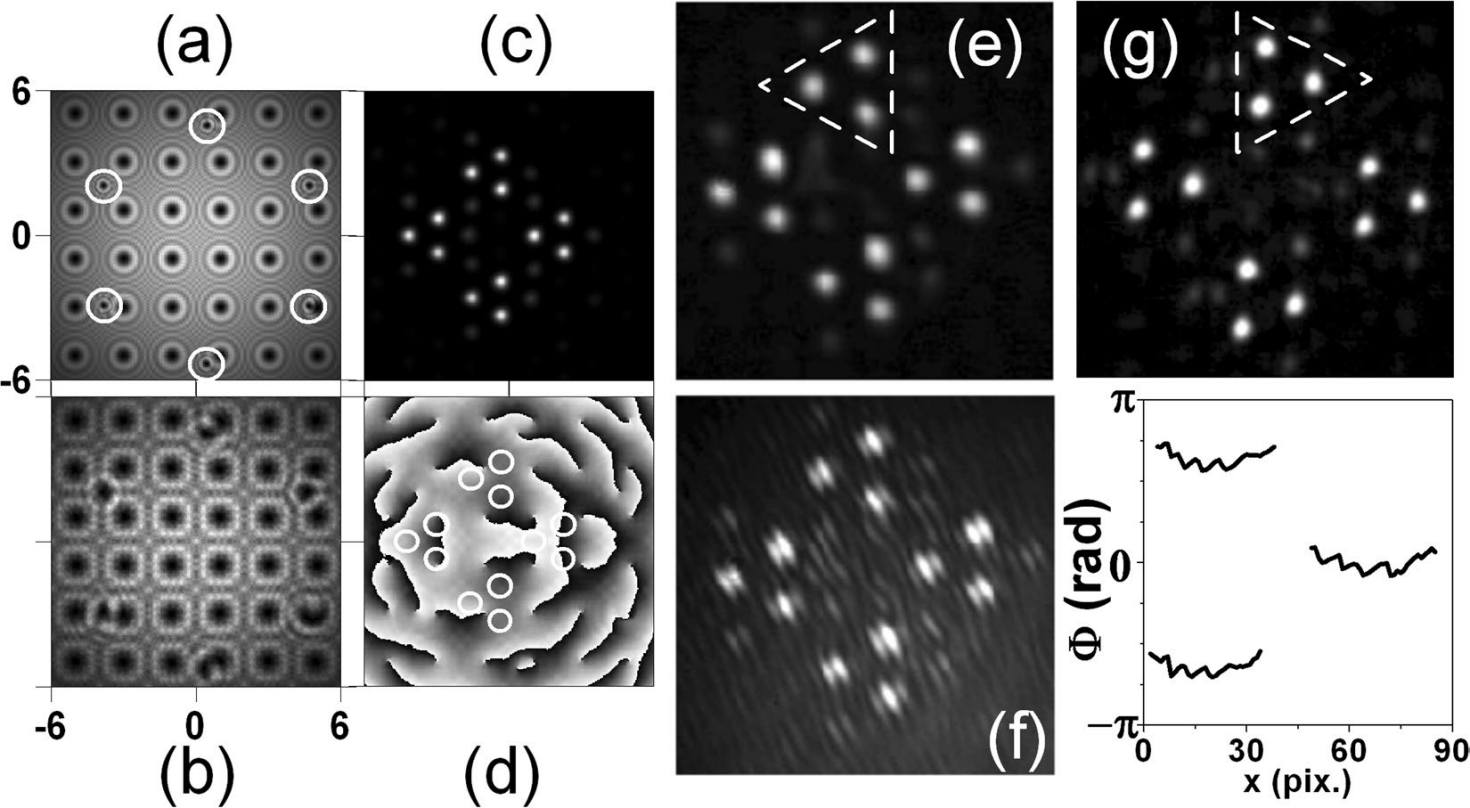
Numerical simulations (**a**–**d**) and experimental results (**e**–**g**). Square-shaped OV array with a lattice constant 41 pix. is encoded on *SLM*1. Hexagonal OV lattice with lattice node spacing of 101 pix. is encoded on *SLM*2. Intensity distribution just behind *SLM*2 (**a**) and in front of the focusing lens *L* (**b**). Calculated far-field intensity distribution (**c**) and its respective phase profile (**d**). Experimentally recorded intensity distributions (**e**,**g**), interference pattern (**f**), and retrieved phase profiles (graph) of the bright peaks denoted in frame (**g**) for the mixed lattices for the same ratio between their lattice constants. Dashed triangle - small-scale structure discussed in the text.

In Fig. 3 we show numerical simulations (a–d) and experimental results (e–g) for the case of mixing square-shaped OV array (lattice constant Δ_*sq*_ = 41 pix.) with a hexagonal OV lattice with lattice node spacing of Δ_*hex*_ = 101 pix. In the particular simulation, the square array is encoded on *SLM*1 and the hexagonal one is encoded on *SLM*2. In frame (a) the OVs constituting one elementary cell of the hexagonal lattice are encircled. In this way we show the large ratio between the node spacings of both lattices and the broadening of each OV of the square lattice propagating from *SLM*1 to *SLM*2 as compared to the width of the newly-born OVs from the hexagonal lattice just after *SLM*2. All OVs continue to propagate in free space (and to broaden due to the diffraction) to the focusing lens *L* (frame (b)). In frame (c) we show the calculated far-field intensity distribution of the mixed square-shaped and hexagonal OV lattices as well as the respective phase profile (frame (d)). As seen in frame (c), the *small-scale* structure is the one of the hexagonal OV lattice with the larger node spacing. There are three dominating peaks situated in the apices of an equilateral triangle, which is inscribed in a rotated triangle-like structure of gradually less intense beams. Four such small-scale structures appear arranged in the apices of a rhomb thus forming the *large-scale* structure determined by the square OV array with the smaller lattice constant. The notations *small-scale* and *large-scale* structures are of course arbitrary and depend on the ratio between the lattice constants of the respective lattices. Nevertheless, the effect is a beautiful manifestation of the two known features of the Fourier transformation performed by a thin lens, namely:

i. the Similarity theorem stating that “wide” functions in the time (space) domain correspond to “narrow” functions in the (spatial) frequency domain.
ii. and the Convolution theorem stating that the Fourier transform of the product of two integrable functions is given by the convolution of their Fourier transforms.

Experimentally recorded intensity distributions (frames (e, g)) and interference pattern (frame (f)) of the mixed lattices are shown in Fig. 3 too. For better visualization we highlighted the small-scale structures in frames (e, g) with a dashed triangle. The same ratio of their lattice constants is used in the numerical simulations. As seen in frame (e), the experimental data perfectly match the numerical ones (frame (c)). Moreover frame (g) in Fig. 3 is confirming experimentally, that the small-scale triangular structures of sub-beams can be rotated by 180° by inverting the signs of all TCs of the OVs constituting the hexagonal lattice or by changing the order of the creation of the individual lattices on the SLMs. The detailed inspection of the encircled portions of the numerically obtained phase profile given in frame (d) shows that all bright sub-peaks have flat phases. This is in agreement with the observed parallel interference stripes in the experiment (frame (f)) when the beam in the reference arm of the interferometer (Fig. 1) is slightly inclined with respect to the beam in the object arm. In the graph in Fig. 3 we present horizontal phase cross-sections of the peaks composing the small-scale structures marked with dashed triangle in frame (g). These cross-sections obtained by the four-frame technique for interferogram analysis^28,29^ confirm the flat phases of the bright peaks. The small curvature of the phase profiles in the graph in Fig. 3 is probably due to a small offset of the CCD-camera chip from the focal plane of the lens.

**Figure 4:**
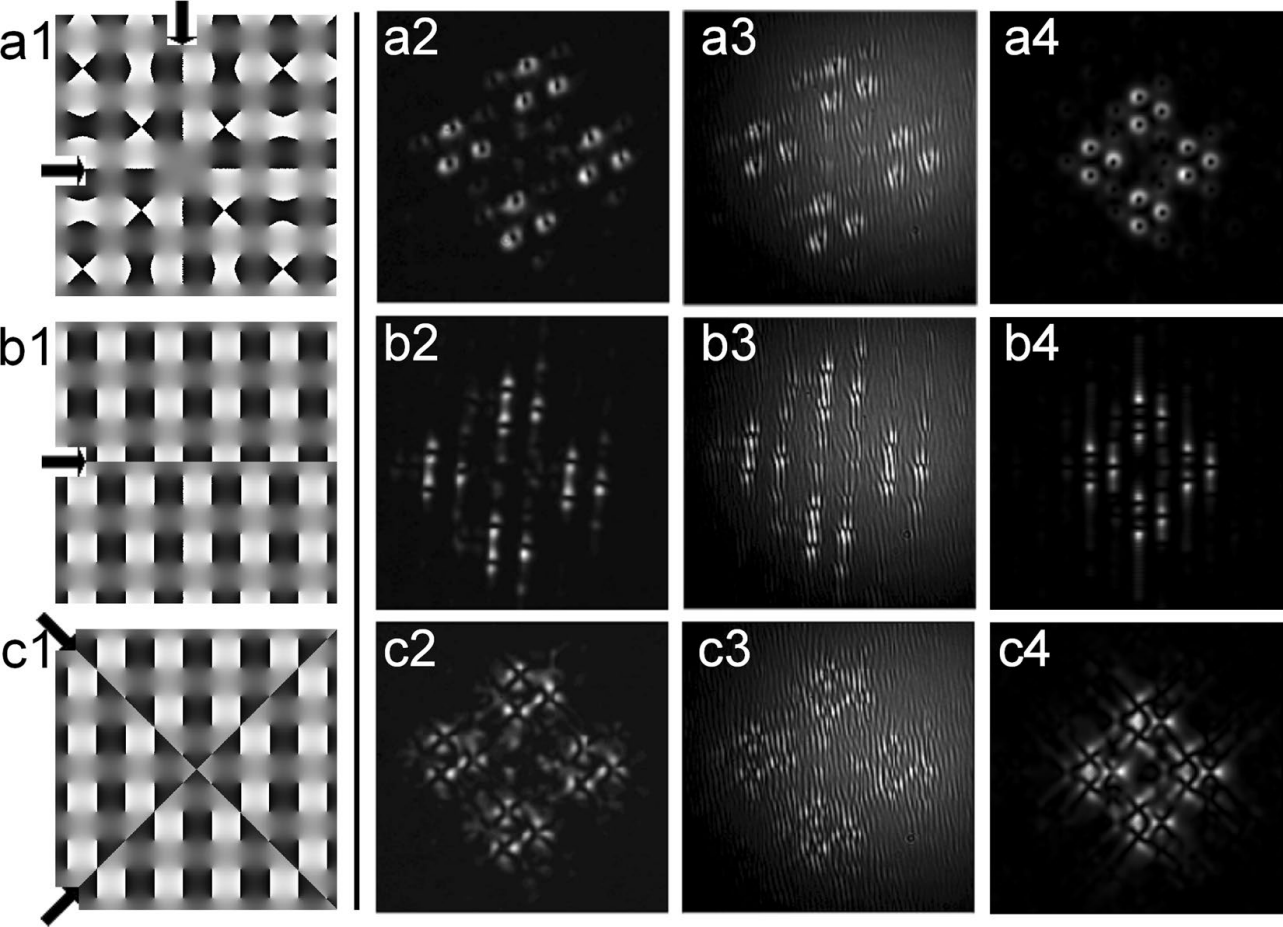
Left: Phase distributions of portions of square-shaped OV lattices with a removed central OV (**a1**), and with an encoded 1-D (**b1**) and quasi-2-D phase dislocations (**c1**). The arrows point to the position of the respective phase dislocation. Right: Additional structuring of the far field intensity profiles of the mixed square/hexagonal-shaped OV lattices by removing an OV (**a2**–**a4**), and by adding 1-D dark beam (**b2**–**b4**), and a quasi-2-D dark beam (**c2**–**c4**). First column of frames (**a2**,**b2**,**c2**) - experimental intensity profiles, second column - recorded interferograms, last column - numerical results. Lattice node spacings on the SLMs: Δ_*sq*_ = 41 pix. for the square-shaped and Δ_*hex*_ = 101 pix. for the hexagonal OV lattice. In each frame in the last column we show 10% of the total computational area.

In this way we arrived at the idea to also use the Convolution theorem of the Fourier transformation for additional structuring of the generated sub-beams. We remind ourselves that an OV nested symmetrically on its host beam remains an OV in the focus of the lens. In the left three phase distributions in Fig. 4 we present some 10% of the calculated and used phase profiles of square OV lattices with a removed OV (plot (a1)), and with an additionally encoded 1-D (plot (b1)) and quasi-2-D phase dislocation (plot (c1)). The arrows in this left panel of plots are intended to guide the eye to their respective positions. In contrast to the truly 2-D point phase dislocation carried by an OV, the one-dimensional (1-D) phase dislocation is just a *π*-phase step along a line (Fig. 4, frame (b1)) resulting in an 1-D dark beam (see Figs 1 and 2 in^30^) while the quasi-2-D dislocations are crossed 1-D phase steps (Fig. 4, frame (c1); see also Fig. 3 in^31^). In Fig. 4(a2,b2,c2) we present measured far-field images resulting from combinations of the mixed OV lattices and an OV (Fig. 4(a2)), a 1-D spatial phase dislocation (Fig. 4(b2)), and a crossed 1-D (i.e. quasi-2-D) spatial phase dislocation (Fig. 4(c2)). Indeed, we can generate a rhomboidal large-scale structure consisting of sub-beams ordered in triangles with an OV nested in each of the 12 bright sub-peaks (see Fig. 4, row (a2–a4)). To this end, a single OV with opposite TC is added to (equivalently - removed from) the phase corresponding to the large square-shaped OV lattice. Using the same approach, we encoded also 1-D and quasi-2-D spatial phase dislocations in the generated sub-beams in the far-field (Fig. 4, rows (b2–b4) and (c2–c4), respectively). The particular position of the 1-D phase dislocation in the square-shaped lattice shown in frame (b1) of Fig. 4 is symmetrical with respect to the two nearest rows of OVs. Numerical calculation and measurements for other positions including the situation of 1-D dislocation crossing a row of OVs showed the same power density distribution (frame (b4)). The same was observed when the quasi-2-D phase dislocation was shifted with respect to the position marked in frame (c1) in Fig. 4. We attribute this result to the known feature of the Shift theorem of the Fourier transformation: Shift in the spatial domain results in a linear phase term in the spatial frequency domain.

In column (a3–c3) of Fig. 4, we present the respective experimentally recorded interference patterns. In frame (a3) upward interference lines splittings can be recognized confirming that all 12 OVs nested on the bright sub-beams have the same topological charges. In Fig. 4(b3,c3) the interference lines are shifted along a line (b3) or along two crossed lines (c3) by half a period, thus indicating 1-D and quasi-2-D phase shifts of *π* and, hence, confirming the presence of spatial phase singularities. We confirmed that the results remain similar after changing the OV lattice node spacings while keeping the square-to-hexagonal lattice node spacing ratio Δ = Δ_*sq*_/Δ_*hex*_ less than unity. For the data shown in Fig. 4 Δ = 0.41. Qualitatively similar results were recorded for four additional values of Δ ranging from 0.44 to 0.54. The TCs of the OVs placed in the bright focal peaks in row (a1–a4) in Fig. 4 can be reversed e.g. by erasing an OV with an opposite TC (in our experiments - from the square-shaped vortex arrays). In frames (a4, b4, c4) in Fig. 4 we show the respective computational results obtained for Δ = 0.41. As seen, the simulated additional pattern structuring by adding OVs or 1-D dark stripes is in excellent agreement with the experiment. The numerical data in the last case of structuring the peaks with quasi-2-D dark beams also agree well with the experiment, however in both cases the beam’s fine sub-structures appear closely spaced and partially overlap. This can be improved by optimizing Δ = Δ_*sq*_/Δ_*hex*_.

**Figure 5:**
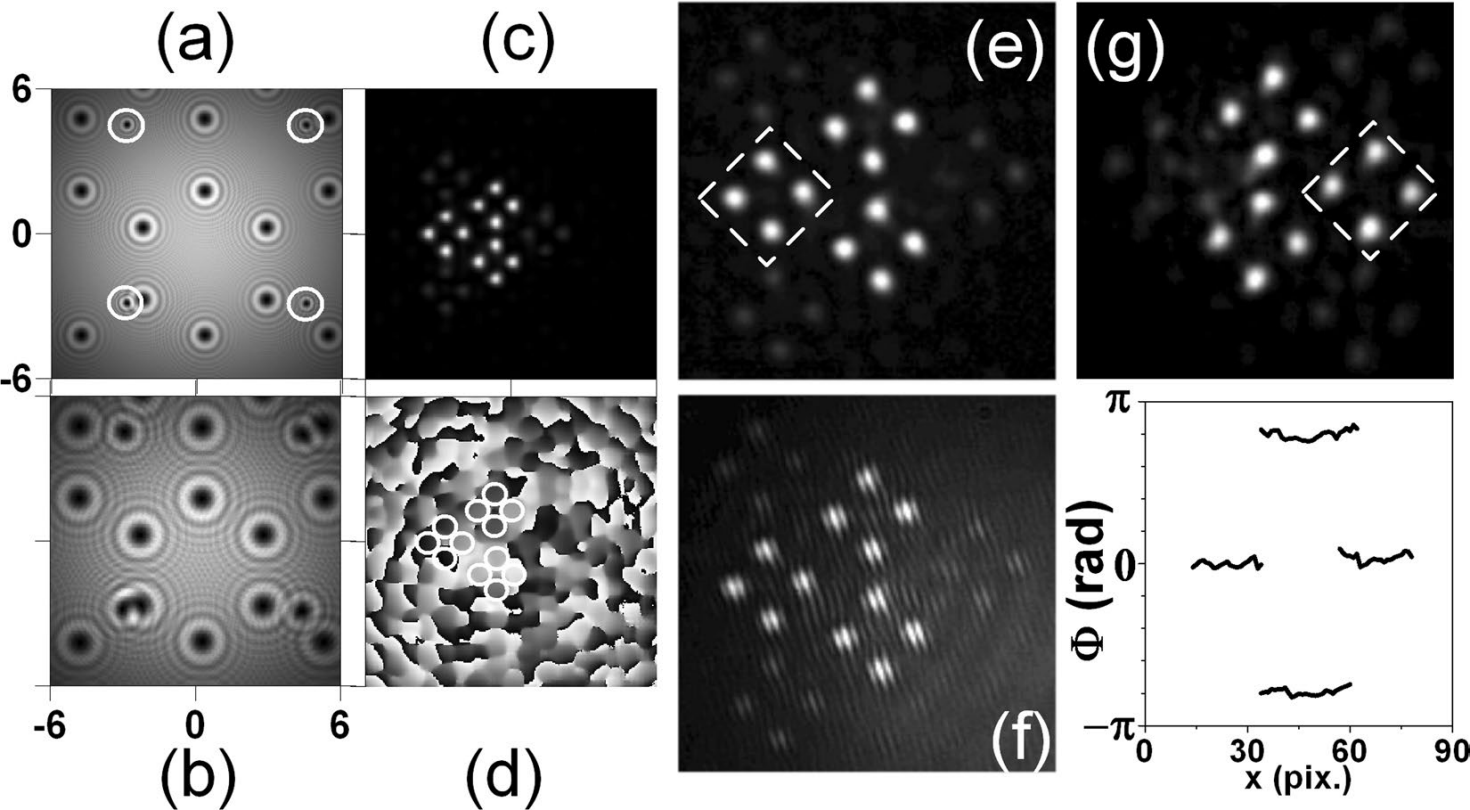
Numerical simulations (**a**–**d**) and experimental results (**e**–**g**). A hexagonal OV array with a lattice constant of 61 pix. is encoded on *SLM*1 and a square-shaped OV lattice with lattice node spacing of 151 pix. on *SLM*2. Intensity distribution just behind *SLM*2 (**a**) and in front of the focusing lens *L* (**b**). Calculated far-field intensity distribution (**c**) and its respective phase profile (**d**). Experimentally recorded intensity distributions (**e**,**g**), interference pattern (**f**) and retrieved phase profiles (graph) of the bright peaks denoted in frame (**g**) of the mixed lattices for the same ratio between their lattice constants. Dashed rhombs - small-scale structures discussed in the text.

Following the style of presentation used in Fig. 3, we show numerical simulations (a–d) and experimental results (e–g) for the case of mixing a square-shaped OV array with a lattice constant of Δ_*sq*_ = 151 pix. with a hexagonal OV lattice with a lattice node spacing of Δ_*hex*_ = 61 pix. in Fig. 5. The well-pronounced difference between these two figures (compare, e.g., frames (c) and (e) of Figs 3 and 5) is due to the fact that Δ = Δ_*sq*_/Δ_*hex*_ = 0.41 for Fig. 3 but Δ = 2.48 for Fig. 5. In Fig. 5(a) the OVs constituting one elementary cell of the square lattice are again encircled in order to emphasize the large ratio between the node spacings of both lattices and the broadening of each of the OVs of the hexagonal lattice on propagation from *SLM*1 to *SLM*2 as compared to the width of the newly-born OVs of the square-shaped lattice just after *SLM*2. All OVs continue to propagate in free space and broaden due to diffraction until they reach the focusing lens *L* (frame (b)). In frame (c) we show the calculated far-field intensity distribution of the mixed square-shaped and hexagonal OV lattices for Δ = 2.48 and the respective phase profile (frame (d)). In contrast to the results for Δ = 0.41 shown in Fig. 3, the small-scale structure in frame (c) of Fig. 5 is formed from the square OV lattice with a larger node spacing (151 pix.) - peaks arranged in the corners of three rhombs, whereby the rhombs are arranged in the apices of an equilateral triangle (Fig. 5, frame (e), dashed rhomb). The triangular large-scale structure is inscribed in a rotated triangle-like structure of much less intense beams. Once again here we see clear manifestation of both Similarity and Convolution theorems for the Fourier transformation. Experimentally recorded intensity distribution (frame (e)) and interference pattern (frame (f)) of the mixed lattices for the same ratio (Δ = 2.48) between their lattice constants are shown in Fig. 5 too. As seen in frame (e), the experimental data perfectly match the numerical ones (frame (c)). It was confirmed experimentally (frame (g)) that the large-scale triangular structure of rhomboidal beam sub-structures can be rotated at 180° by inverting the signs of all TCs of the OVs of the hexagonal lattice or by changing the order of the creation of the individual lattices on the SLMs. A close inspection of the encircled portions of the numerically obtained phase profile given in frame (d) shows that all bright sub-peaks have flat phases, which is unfortunately not obvious without image magnification. This is, however, in agreement with the observed parallel interference stripes in the experiment (frame (f) in Fig. 5) and with the phase cross-sections of the peaks marked in frame (g). The data are obtained by the four-frame technique for interferogram analysis (last graph in Fig. 5).

**Figure 6:**
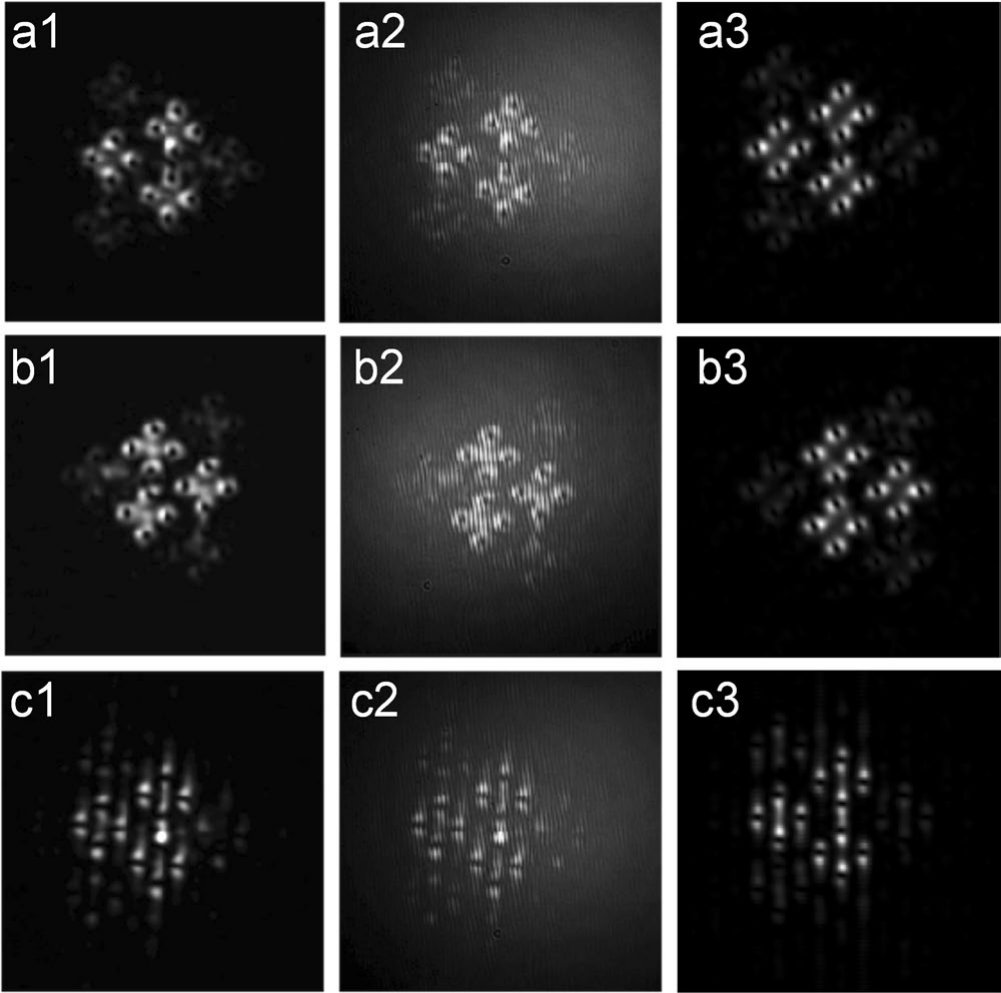
Additional structuring of the far field intensity profiles of mixed square-shaped and hexagonal OV lattices by adding an OV with an opposite TC (**a1**–**a3**) (effectively - by removing it), by changing the order of the generation of the respective OV lattices on the SLMs (**b1**–**b3**), and by adding a 1-D dark beam (**c1**–**c3**). First column of frames - experimental intensity profiles, second column - recorded interferograms. The lattice node spacings as encoded on the SLMs are Δ_*sq*_ = 151 pix. and Δ_*hex*_ = 61 pix. for the square-shaped and for the hexagonal OV lattices, respectively (Δ = 2.48). For the numerical data (last column showing some 10% of the total computational area) again Δ = 2.48, however Δ_*sq*_ = 101 pix. and Δ_*hex*_ = 41 pix.

In contrast to Fig. 4, in which the large-scale structure is a rhomb, in Fig. 6 we demonstrate a triangular large-scale structure. In Fig. 6 we show results confirming the possibility for additional structuring the far field intensity profiles of the mixed square-shaped and hexagonal OV lattices. This was done by adding an OV with an opposite TC (a1–a3), by additionally changing the order of the generation of the respective OV lattices on the SLMs (b1–b3), and by adding a 1-D dark beam (c1–c3). The first column in Fig. 6 shows measured far-field intensity beam profiles. In the second column, using the same ordering, we show the respective experimentally recorded interference patterns. In frames (a2) and (b2), downward and upward fork-like splittings of interference lines can be recognized confirming that: *i*) all 12 OVs nested on the bright sub-beams have the same topological charges and *ii*) all topological charges reverse in sign when the ordering of the phases projected on the SLMs is reversed or by deleting an OV with an opposite TC from the input phase structure (Fig. 4(a1)). The data prove also that when the ordering of the phases projected on the SLMs are reversed, the orientation of the large-scale triangular structure consisting of rhomboidal beam ensembles carrying OVs changes its orientation from (arbitrary) left orientation (frames (a1–a3)) to right orientation (frames (b1–b3)). In frame (c2) the interference lines are shifted along a line by a half a period, which is a clear indication for a 1-D phase shift of *π* confirming the presence of spatial phase dislocation. The experimental results presented in Fig. 6 refer to Δ = 2.48 and remained qualitatively similar to these for square-to-hexagonal lattice node spacing ratio Δ = 3.73. The data shown in Figs 4 and 6 are directly comparable in spatial extent. The numerical data shown in the last column of frames in Fig. 6 refer again to Δ = 2.48, however, different from the case shown in Fig. 5, here Δ_*sq*_ = 101 pix. and Δ_*hex*_ = 41 pix.

**Figure 7:**
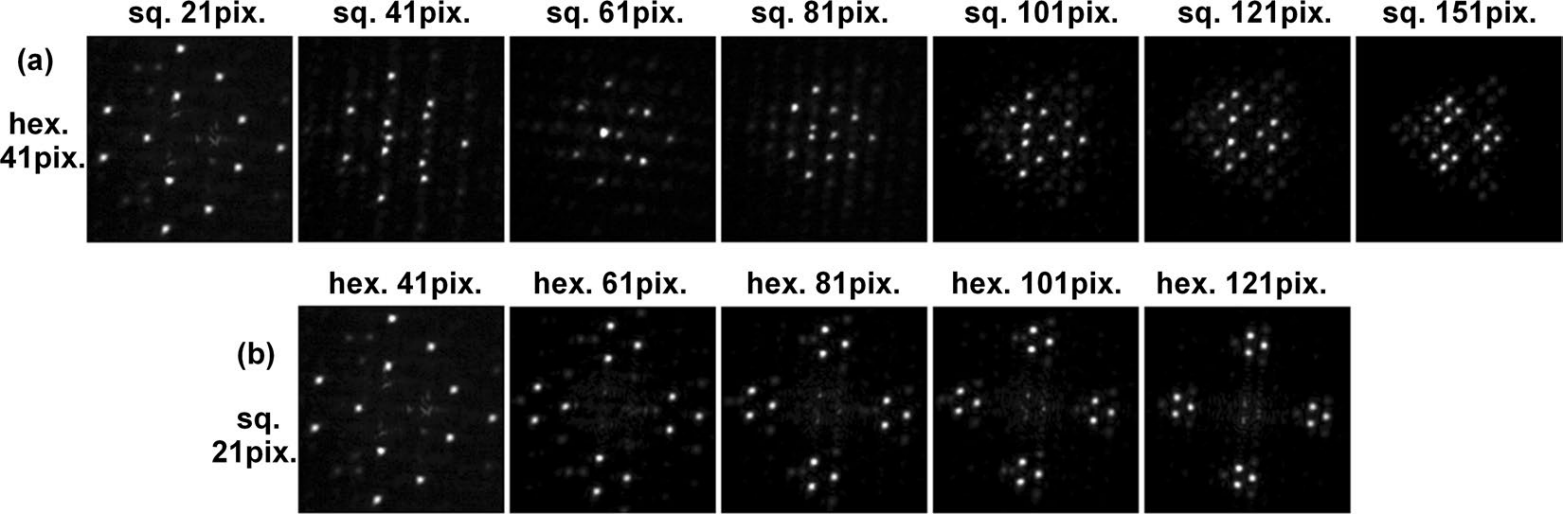
Experimentally-recorded far-field beam reshaping by varying the node spacing of the square-shaped (row (**a**)) or of the hexagonal OV lattice (row (**b**)). In case (**a**) the hexagonal lattice node spacing is Δ_*hex*_ = 41 pix. and Δ = Δ_*sq*_/Δ_*hex*_ varies between 0.5 and 3.7. In case (**b**) Δ_*sq*_ = 21 pix. and Δ varies between 0.5 and 0.17.

**Figure 8:**
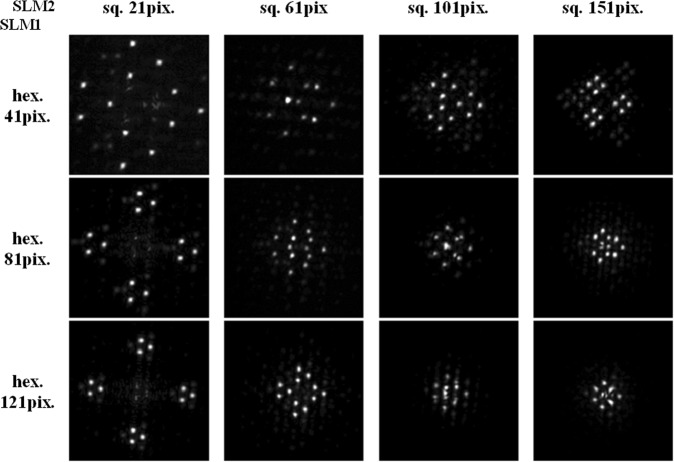
Experimentally-recorded far-field bright beam patterns for different square-to-hexagonal OV lattice node spacings Δ = Δ_*sq*_/Δ_*hex*_ encoded on the SLMs. The top row of frames corresponds to Fig. 7(a), the left column of frames - to Fig. 7(b).

In Fig. 7 we show different experimentally recorded far-field bright beam intensity distributions obtained by varying the node spacing Δ_*sq*_ of the square-shaped (row (a)) or Δ_*hex*_ of the hexagonal OV lattice (row (b)). These data are selected from a larger set of measurements demonstrating the change in the symmetry and in the size of the far-field beam structures by changing the vortex-to-vortex node spacing of one of the lattices, keeping the respective spacing for the other lattice unchanged. In case (a), the hexagonal lattice node spacing is Δ_*hex*_ = 41 pix. One can clearly see that for Δ_*sq*_ = 21 pix. (row (a), left frame) the far-field beam profile has the *small-scale structure* resembling an equilateral triangle with three dominating peaks situated in its apices. In other words, the *small-scale* structure is the one of the hexagonal OV lattice, which has a two-fold larger node spacing, i.e. Δ_*hex*_ = 41 pix. The *large-scale* focal structure resembles a rhomb with the aforementioned triangular small-scale structures in its apices. Thus the *large-scale* structure is the one formed by the square OV lattice with the *smaller* node spacing Δ_*sq*_ = 21 pix. In the other limiting case shown in the most right frame of Fig. 7(a), Δ_*hex*_ = 41 pix. is kept unchanged and Δ_*sq*_ is increased from 21 pix. to 151 pix. The change in the symmetry of the structure is impressive. For Δ_*sq*_ > 80 pix. the *small-scale* structure becomes rhomboidal and the peaks arranged in the apices of the rhombs are ordered in a triangle-like *large-scale* structure. In other words, in this limiting case the *small-scale* structure is this of the square lattice with the *larger* Δ_*sq*_ = 151 pix., whereas the *large-scale* structure is formed by the hexagonal lattice with the *smaller* Δ_*hex*_ = 41 pix. The transition from one symmetry of the distribution of the bright peaks to another symmetry seems to happen (Fig. 7, row (a)) approximately when Δ_*hex*_ = 41 pix. and Δ_*sq*_ = 61 pix. At equal node spacings the large-scale structure can be still recognized to be this of the square lattice. For Δ_*sq*_ = 81 pix. it is already changed to the form coming from the hexagonal lattice. The further increase of the node spacing of the square lattice shows well pronounced shrinking of the large-scale triangular structure. The frames shown in row (b) in Fig. 7 provide a nice visualization of the mentioned Similarity theorem for the Fourier transformation: For Δ_*hex*_ > Δ_*sq*_, the increase of the node spacing of the hexagonal lattice Δ_*hex*_ from 41 pix. to 121 pix. results in a shrinking of the focal *small-scale* structures. It is worth noting that the “centers of mass” of the four small-scale triangle-like structures do not change their mutual positions because Δ_*sq*_ remains the same. Figure 8 is intended to provide more insight into the dynamics shown in Fig. 7. The results in Figs 7 and 8 clearly indicate that Δ = Δ_*sq*_/Δ_*hex*_ could serve as a control parameter for generating the desired focused structure.

## Conclusion

In this work we show significant far-field beam reshaping by mixing square-shaped and hexagonal optical vortex lattices. Each of the singular lattices used here is composed of vortices with alternating TCs. We showed that the *small-scale structure* of the observed pattern is a result of the OV lattice of *larger* array node spacing and, conversely, that the OV lattice with *smaller* array node spacing determines the *large-scale structure*. The orientation of the mixed far-field structures is proven to rotate by 180° when all TCs are inverted. We succeeded to additionally host singly-charged OV, 1-D or quasi-2-D singular beams in each of the bright peaks of the mixed far-field patterns by erasing the TC of an OV of one of the arrays or by adding to it the respective singular phase profile of the aforementioned 1-D and quasi-2-D singular beams. We attribute the somewhat “noisy” intensity dots in the experimentally generated focal bright structures to the different sizes of the OV cores of the mixing lattices and to the different reshaping of the neighboring part of the background beams due to the diffraction. This could be improved by imaging the first SLM onto the second modulator and would even enable the use one SLM only. The detailed experimental data for the evolution of the far-field patterns at different square-to-hexagonal vortex array node spacings Δ = Δ_*sq*_/Δ_*hex*_ shows that this quantity could serve as a control parameter for generating the desired focused structure. It will be intriguing to extend the presented approach to other shapes of lattices, e.g. rhomboidal OV lattices, periodic kagome lattices, and to quasi-periodic patterns (Penrose, decagonal; see the systematic description in^12^). These results, in addition to the previously published ones^11^, may appear particularly interesting, as a new degree of freedom, for modifications in stimulated emission depletion (STED) microscopy, for extending the possibilities of generating singular higher-order vector fields^15^, for controllable writing of parallel optically-induced waveguide structures e.g. in (photorefractive) nonlinear media (see, e.g.^14^,) and may appear applicable for orbital angular momentum multiplexing of information^16^ for data transfer using complex optical fields^17–21^.

## Data Availability

The datasets generated during and analyzed during the current study are available from the corresponding author on reasonable request.
